# Case of Conception before the Appearance of the Menses

**Published:** 1852-09

**Authors:** Wm. T. Taylor

**Affiliations:** Philadelphia


### *Case of Conception before the Appearance of the Menses.*

By Wm. T. Taylor, M. D., Philadelphia.

To the Editors of the Medical Examiner.

Gentlemen,—The general experience of the medical world has established it as a physiological fact, that conception cannot take place prior to the appearance of the menses. But there are instances of females becoming mothers, who have never menstruated; they, however, must have conceived just at the time when the catemenia were about to be established, which after parturition and suckling probably occurred regularly.

The following case, which I have met with, seems to be an anomaly in the annals of midwifery:

During the month of June, 1851, I was requested to visit Hannah B., a mulatto, who was pregnant with an illegitimate child. Was much surprised at finding my patient herself was a mere child in appearance and manner. She was thirteen years of age on the 3d of the previous February, and, though she had never menstruated, was, when I saw her, in the eighth month of gestation. Her general condition was plethoric; her breasts well developed, and the areola quite dark.

On the 13th of August she was taken in labor, but in consequence of a prolapse of the funis umbilicalis, which could not be replaced, I delivered her of a still-born child, of the usual size, and perfectly formed. The lochia continued for a few days, and she passed through the accustomed period after delivery very favorably.

It is now one year since, and her menses have not yet made their appearance, nor has their been any vicarious discharge; her health during the whole time remaining perfect.

Never having read of a case of the kind, I have sent it to you for publication, should you think it of sufficient interest.

*Philadelphia, August 19th*, 1852.

